# Non-invasive vagus nerve stimulation for the treatment of acute asthma exacerbations—results from an initial case series

**DOI:** 10.1186/1865-1380-6-7

**Published:** 2013-03-19

**Authors:** Elmin Steyn, Zunaid Mohamed, Carla Husselman

**Affiliations:** 1 Trauma Centre, Christiaan Barnard Memorial Hospital, 181 Longmarket Street, Cape Town, 8001, South Africa; 2 Mediclinic Cape Gate Hospital, Cnr Okavango and Tanner Roads, Brakenfell, Cape Town, 7560, South Africa; 3 Netcare Kuilsriver Hospital, 33 van Riebeeck Rd, Kuils River, Cape Town, 7580, South Africa

**Keywords:** Bronchoconstriction, Non-invasive vagus nerve stimulation, Acute asthma, Clinical study, Device, Acute exacerbation of asthma

## Abstract

**Abstract:**

A prospective multicentre clinical study was initiated to evaluate the safety and potential clinical benefit of non-invasive vagus nerve stimulation (nVNS) for the treatment of bronchoconstriction exacerbations in asthmatics. Due to slow enrolment and design changes of the device, the study was prematurely terminated after enrolment of four eligible patients. Three of the four patients were considered treatment successes based on improvement in FEV_1_, improvement in VAS dyspnoea scoring, and the absence of device-related adverse events.

**Trial Registration:**

ClinicalTrials.gov Identifier: NCT01385306

## Findings

Asthma remains a serious global health issue placing a major burden on health care resources despite the progress made in the methods of treatment and diagnosis of the disease [1,2]. The majority of treatment options for asthma management are pharmacological; however, a recent publication describes positive findings with the use of a novel percutaneous vagus nerve stimulator for treatment of acute severe asthma exacerbations [3]. In this letter, the authors report their early experiences with an nVNS device for the relief of acute bronchoconstriction in asthmatics treated in an emergency department setting.

Independent ethics review board approval was granted for the prospective multicentre study (South African Medical Association Research Ethics Committee: Protocol No. BC-SA-01) and informed consent was obtained for all study subjects. Subjects between the ages of 18 and 70 years were recruited from the pool of patients admitted to the emergency departments at participating investigational sites with a diagnosis of bronchoconstriction due to asthma. Enrolment criteria included a VAS dyspnoea score of 3 or more with a failure to show at least a 1.5 point improvement and an FEV_1_ of less than 60% predicted, following SOC treatment [SOC treatment included supplemental oxygen, inhaled β_2_ agonists (without ipratropium), and/or inhaled corticosteroids]. Subjects received a total of two nVNS treatments with the study device, 30 min apart and each lasting 60 s. During this period, subjects continued to receive standard pharmacological treatment consistent with the standard of care at their institution as required. Subjects were monitored at regular intervals to determine changes in respiratory status and vital signs for 90 min post initial nVNS. The success criteria for the primary outcome measurements were an improvement of a minimum of 12% above baseline in FEV_1_, an increase of at least 1.5 points over baseline in the VAS dyspnoea score immediately after the second nVNS and the absence of any serious device-related adverse events.

**Figure 1:**
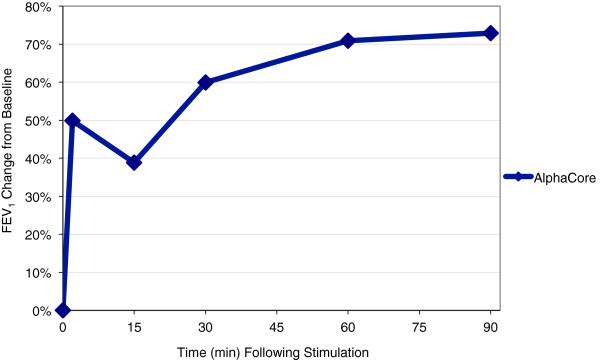
**Effect of vagal nerve stimulation on FEV**_**1 **_**(*n *= 4).**

**Figure 2:**
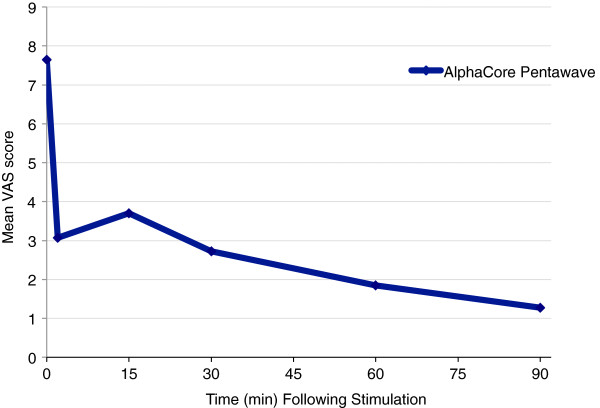
**Effect of vagal nerve stimulation on work of breathing (VAS) (*n *= 4).**

Since study enrolment was significantly slower than anticipated and a next-generation device had become available before enrolment could be completed; the decision was taken to terminate the study after 4 eligible patients out of the originally planned 25 patients had been treated. However, findings for these patients were noteworthy and are summarised in Table 1, Figures 1 and 2. Three subjects were classified as treatment successes based on the predefined definition. One subject satisfied the VAS dyspnoea score and adverse event criteria for success, but only achieved an increase of 9% in FEV_1_. FEV1 in the four treated subjects showed a mean improvement of 73% increase over baseline at 90 min post-stimulation; similarly, mean VAS decreased from a score of 8 pre-stimulation to 1 at 90 min. The mean heart rate was 106 pre-nVNS, with a decrease to 85 at 90 min post-nVNS. Mean systolic blood pressure increased by 4% after the first nVNS, but decreased at 60 and 90 min post-nVNS to lower levels than pre-nVNS. One patient experienced an adverse event, a previously diagnosed respiratory tract infection requiring antibiotic treatment on admission to the unit, unrelated to the device or procedure.

**Table 1:** **Table showing the effect of nVNS on FEV1 (mean change from baseline) and VAS (*n*= 4)**

**Time**	**FEV1**	**VAS**
0	0	7.65
2	50%	3.08
15	38.80%	3.70
30	59.94%	2.73
60	70.91%	1.85
90	72.90%	1.28

Although the traditional view has held that stimulation of the vagus nerve results in increased airway resistance and decreased compliance [4,5], an electrical stimulus signal that reduced histamine-induced bronchoconstriction in guineapig and swine has recently been described [6]. This is a relatively low amplitude signal, significantly below the threshold required to stimulate efferent vagus fibres that would evoke bronchoconstriction or bradycardia [6]. Our results support the recent findings of Miner et al., who demonstrated improvements in FEV1 and work of breathing, little impact on haemodynamic stability, and the absence of serious adverse events when using a percutaneous device for vagal nerve stimulation in 25 patients with asthma exacerbations reporting to the emergency department [3].

From a user perspective, the non-invasive device has obvious advantages over the percutaneous device, which requires experience with both the placement of central venous lines and the possibility of the occurrence of concomitant adverse events. Furthermore, compared to the traditional pharmacological treatment, there are several potential benefits of using a non-invasive device with the possibility of eliminating side effects associated with the use of drugs. The outcomes presented from the cases treated in this report suggest that nVNS may be a feasible nonpharmacological treatment option for the management of acute asthma. RCTs of the nVNS device are currently planned.

## Abbreviations

CE: Conformité Européenne (European Conformity); CI: Confidence interval; FEV1: Forced expiratory volume in the first second; nVNS: Non-invasive vagus nerve stimulation; RCT: Randomised controlled trial; SOC: Standard of care; VAS: Visual analogue scale.

## Competing interests

Support for this study was provided by ElectroCore LLC. All authors received payment from ElectroCore LLC for their investigator-related duties.

## Authors’ contributions

ES was the principle study investigator and conceptualised the manuscript, provided input on the drafting of the manuscript and took responsibility for finalising the manuscript. ZM and CH were study investigators, gave input to the drafting of the manuscript, and read and approved the final manuscript. All authors read and approved the final manuscript.

## Authors’ information

ES is Clinical Director of the Trauma Centres at Christiaan Barnard Memorial Hospital and Vincent Pallotti Hospital in Cape Town, South Africa. She is immediate Past President of the Trauma Society of South Africa [MBChB, M.Med (General Surgery), FCS (SA)].

ZM is Head of the Emergency Centre at Mediclinic Cape Gate Hospital in Cape Town, South Africa (MBChB).

CH is in private practice at the Trauma Unit of the Netcare Kuilsriver Hospital in Cape Town, South Africa (MBChB).
